# Correlation of radiographic variables to guide safe implant positioning during acetabular surgery and hip replacement: a retrospective observational study

**DOI:** 10.1186/s13037-019-0192-6

**Published:** 2019-03-12

**Authors:** Simon Tiziani, Georg Osterhoff, Jan-Farei Campagna, Clément M. L. Werner

**Affiliations:** 1 Department for Trauma, University Hospital Zurich, Raemistrasse 100, 8091 Zurich, Switzerland; 2 Balgrist University Hospital Zurich, Forchstrasse 340, 8008 Zürich, Switzerland

**Keywords:** Medial bone stock, Reaming, Acetabular index

## Abstract

**Background:**

Knowledge of periacetabular anatomy is crucial for prosthetic cup placement in total hip arthroplasty and for screw placement in anterior fixation with acetabular fractures. It is known that degree of hip dysplasia correlates with medial bone stock and that medial bone stock shows a weak correlation to Lequesne’s acetabular index (AI). Aim of this study was to investigate a possible correlation between AI and the newly proposed medial safe zone.

**Methods:**

AI and the medial save zone were measured on 419 hips using a computed-tomography scan of the pelvis. AI was assessed on a 2D reconstructed anterior-posterior view of the pelvis using VOXAR™. Correlation was measured using the Pearson correlation coefficient.

**Results:**

Mean AI was 4.2 degrees (SD 4.9 degrees). Mean medial safe zone was 8.1 mm (SD 1.9 mm). There was a significant correlation between AI and medial save space with a Pearson correlation coefficient r = 0.33 (*p* = .001).

**Conclusion:**

There is a weak correlation between AI and medial safe zone. AI should not be used to predict medial safe zone. Due to the weakness in correlation AI is not suited for predicting medial safe zone. However, a low or negative AI can be a warning sign for less medial safe zone, prompting surgeons to take care when reaming in THA or placing periacetabular screws.

## Background

Precise anatomical knowledge of the periacetabular region is required both for total hip arthroplasty (THA) and surgical fixation of acetabular fractures. One of the parameters surgeons are interested in, is the medial bone stock or the thickness of the quadrilateral plate. With THA this information is required for the process of reaming. Both under- and overreaming have been made responsible for acetabular component protrusion into the pelvis [1, 2]. Further, a sparsely present medial bone stock has been named a risk factor for intraoperative medial breach in THA [3]. Similarly, medial bone stock is of interest in the case of open reduction and internal fixation of acetabular to accurately plan screw placement and in avoid intraarticular screw malposition.

As especially with THA to treat osteoarthritis In search of an indicator of medial bone stock on conventional radiographs, in a previous study [4] authors tried to show a correlation between the established coxometric parameters, Lequesne’s acetabular index and Wiberg’s lateral center edge angle and medial bone stock. They were able to demonstrate a weak positive correlation between the acetabular index and the medial bone stock. As the correlation was weak, the authors did not recommend using the acetabular index as a predictor for medial bone stock when planning THA or reconstructive acetabular surgery. Lequesne first described to acetabular index as a measure for acetabular roof inclination in 1963 [5], The acetabular index is valued in degrees to represent sourcil inclination. Increasing acetabular index signifies increasing inclination and vice versa. Values above 12° are considered pathological indicating decreased acetabular coverage, as seen in dysplasia of the hip [5, 6].

As especially for screw placement in reconstructive acetabular surgery, medial bone stock alone is not the deciding factor. Medial bone stock is lowest at the site of the acetabular fossa. Screws that overpenetrate the medial bone stock and come to lie in the acetabular fossa might not interact with the hip joint and thus not cause any problems. The extended range including the medial bone stock and extending up to the articulating surface is therefore also of interest. We therefore introduce the medial safe zone. Which is defined as the shortest distance from the inner edge of the medial bone stock to a circle fitted to the contour of the articulating acetabulum and encircling the femoral head. Everything that lies outside of this circle should not interfere with the hip joint.

The aim of this study was to look for a correlation between Lequesne’s acetabular index (AI) as a measure of acetabular roof inclination and the medial safe space. The hypothesis was that a decreased AI correlates with a decrease in medial safe zone, making patients with a “flat” AI potentially more difficult to operate on.

## Material

### Patients

This study was approved by the local ethics committee (Kantonale Ethikkomission Zürich; KEK-ZH-Nr.2011–0507). For this study, 248 patients (90 female, 158 male) which had undergone a CT scan including the pelvis were identified from a database (most of the scans were whole-body scans in the acute trauma setting). In order to standardize radiographic measurements of the acetabular index (AI), anteroposterior (a.p.) radiographs of the pelvis were rendered form the computed-tomography (CT) data using an imaging software (VOXAR™, TMVS Europe, Edinburgh, UK). Reconstruction of the a.p. views was done in accordance with the criteria set forth by Siebenrock et al. [7]. This method has previously been used to measure parameters design for conventional radiographs on computed-tomography scan data sets [8].

Out of the 496 hips, 79 were excluded due to presence of either a hip or acetabular fracture, total hip arthroplasty, or poor quality of the 2D reconstruction. As the a.p. reconstructions were used to measure the AI, insufficient quality was defined as the inability to differentiate the sourcil from the rest of the acetabulum.

### Lequesne’s acetabular index (Fig. 1)

The acetabular index is the angle between a horizontal line in reference to the pelvis and a line connecting the start and end point of the acetabular roof, the sourcil. For the horizontal line a line through both teardrop figures was chosen. The angle to the inclination of the acetabular roof was than measured using an integrated tool provided by the picture archiving and communication system used (PACS; IMPAX, Agfa, Mortsel, Belgium). Values were noted in degrees rounded to one decimal. Angles facing in cranial direction compared to the horizontal line were regarded as being positive, and angles facing caudally were noted as being negative.

**Fig. 1:**
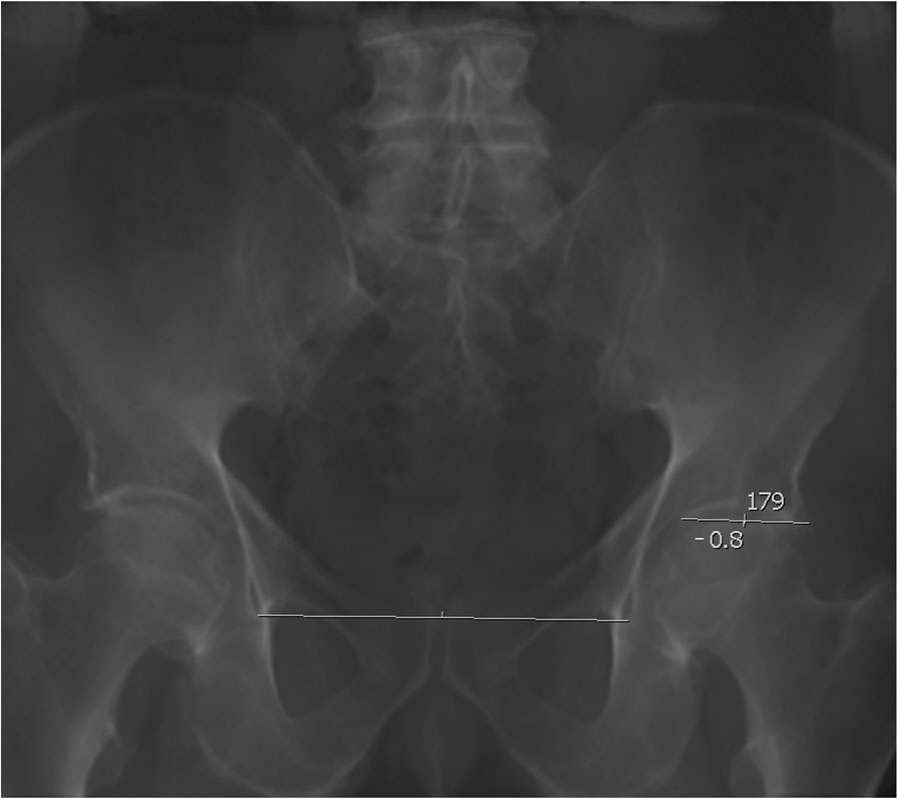
Measurement of the acetabular index on reconstructed 2D anterio-posterior views of the pelvis

### Medial safe zone (Fig. 2)

The medial safe zone was defined as the distance from the medial edge of the medial bone stock up to a circle fitted to the bony acetabulum and encompassing the femoral head. This way, everything which came to lie outside this circle, would not interfere with the hip joint. The distance was measured using the distance measuring tool included in the PACS. The shortest distance between medial bone stock and the circle was chosen. Values were noted in millimetres rounded to one decimal. All measurements were taken by a senior resident (S.T.) under the supervision a senior consultant (C.W.).

**Fig. 2:**
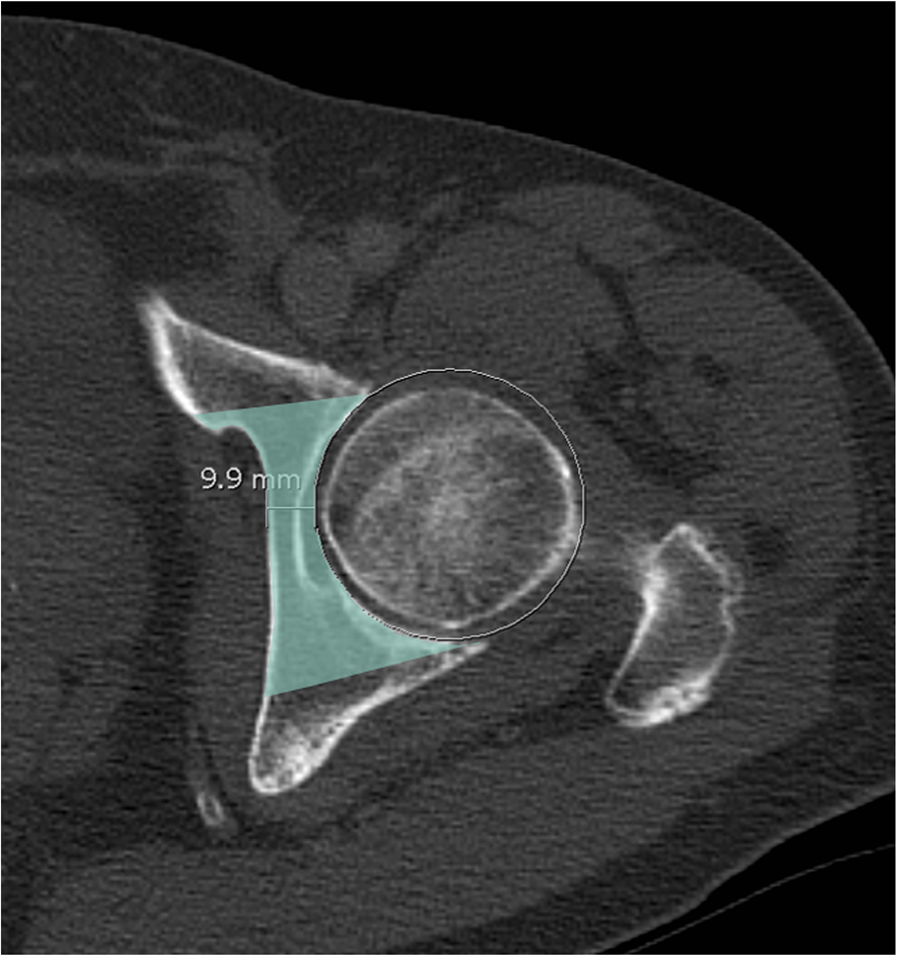
Measurement of the minimal width of the medial safe zone (indicated in color). Circle around the femoral head marking the possible range of interaction in articulation between the femur and a hypothetical acetabular screw

### Statistical analysis

All data was recorded in an Excel database (Microsoft, Washington, USA) and exported to SPSS 23.0 (SPSS Inc., Chicago, USA) for analysis. Histograms were used to determine whether the data was normally distributed. As this was the case and both parameters were continuous, correlation was assessed using the Pearson correlation coefficient.

## Results

In total 419 hips (146 female, 273 male, 17–94 years of age. Mean age 51) were included into the study.

Mean value for acetabular index was 4.2° (range − 10 – 25°, standard deviation 4.9°). The mean medial save space was 8.1 mm (range 3.3 mm – 15.2 mm, standard deviation of 1.9 mm).

**Fig. 3:**
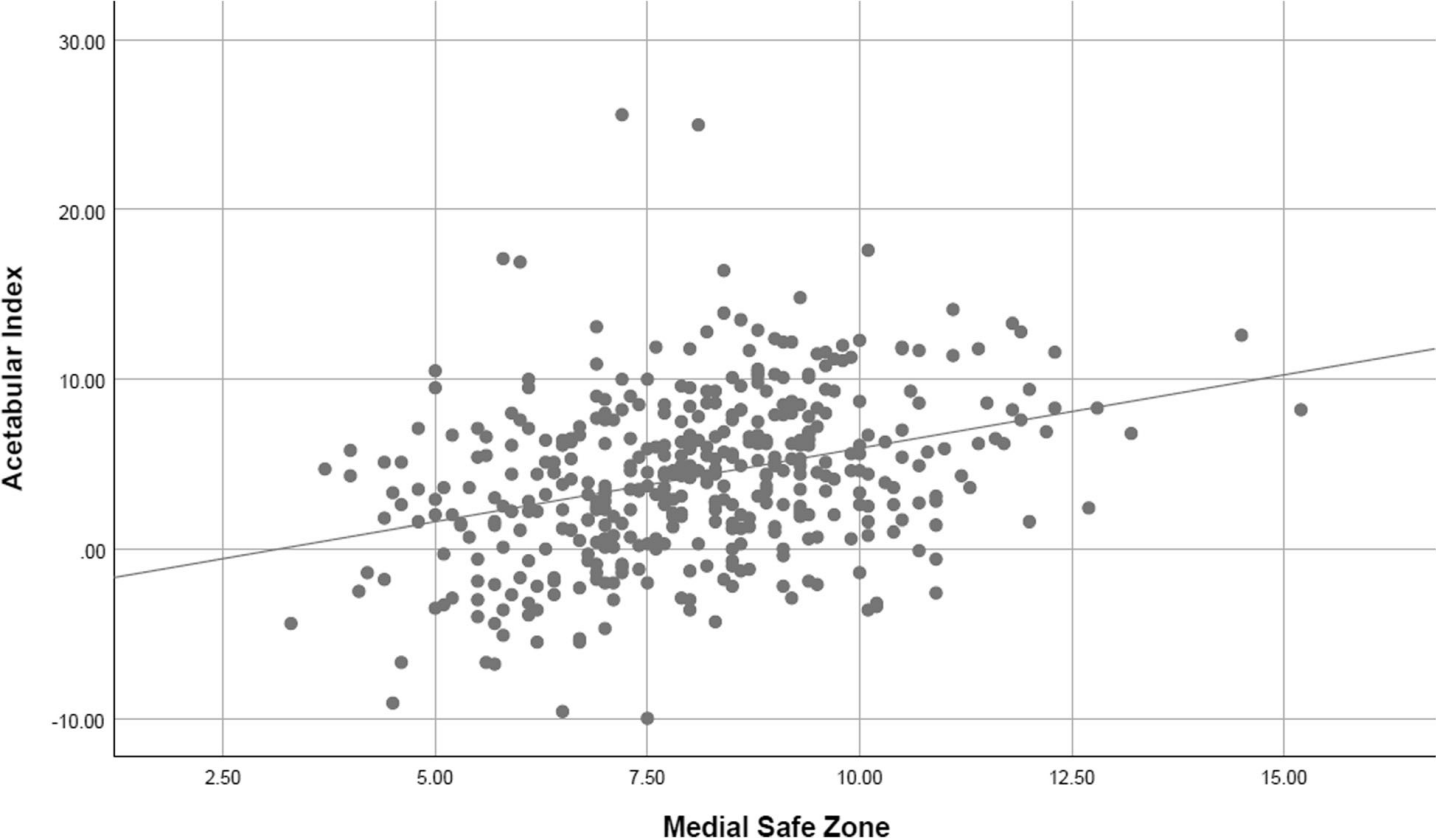
Showing the measured data as a scatter plot with linear regression line. Acetabular index measured in degrees (°) and the medial safe zone in milimeters (mm)

There was a significant but weak correlation between the acetabular index and the medial safe space (*p* = 0.001, Pearson’s *r* = 0.33) (Fig. 3).

## Discussion

Precise knowledge of the periacetabular anatomy is crucial for reaming in THA and during open reduction and internal fixation of acetabular fractures using anterior surgical approaches. It is known that with increasing dysplasia of the hip, medial bone stock increases as well [9]. This, combined with the fact that Lequesne’s acetabular index is used to detect or measure dysplasia of the hip motivated the previous study trying to find a correlation between AI and medial bone stock [4]. The linear correlation that would make it able to predict medial bone stock by looking at the acetabular roof inclination could however not be demonstrated.

The aim of our study was to investigate whether Lequesne’s acetabular index and the medial safe zone showed a significant correlation. Furthermore, if there was a correlation, whether it was strong enough to allow for using AI to predict the medial save zone preoperatively. We could show that AI correlated with the medial save zone. This was a significant positive correlation, meaning that with increasing AI, medial safe zone was to increase as well. However, the correlation coefficient was *r* = 0.33, which amounts to a weak correlation. The results align with the results obtained by Werner et al. in their study correlating acetabular index and medial bone stock at a correlation coefficient of *r* = 0.37.

On the grounds of this weak correlation, we would refrain from suggesting the use of the acetabular index to predict the medial safe zone. However, there are two studies now that suggest one should be careful when reaming in THA or when placing screws in internal fixation of the acetabulum if value for the acetabular index is low or even negative, as medial bone stock and medial save space tend to be sparse as well. A high value for the acetabular index should on the other hand not lead one to assume vast medial bone stock and a lot of medial safe space to work with.

In the case of open reduction and internal fixation of the acetabulum this causes less of a problem, as patient’s routinely receive computer tomography when acetabular fractures or fractures of the pelvic ring are suspected. In the rare case that such fractures are diagnosed on conventional radiographs a preoperative CT scan is usually performed. Preoperative measurement of the medial bone stock or the medial safe zone can then be taken directly form the computed-tomography scan. With THA, especially for treatment of osteoarthritis, preoperative CT scans are not routinely conducted, making medial bone stock or medial safe zone assessment an unsolved issue.

There are limitations to this study. First, many hips had to be excluded because quality of the 2D anterior-posterior reconstruction was too poor. The source of this difference in quality is unclear, leaving room for a bias in patient selection. However, looking at the obtained data for acetabular indices, we find that our measurements are alignment with previously reported normal values for Lequesne’s acetabular index [6]. Another limitation to this study is the lack of intra- and inter-observer control.

## Conclusion

There’s a weak correlation between Lequesne’s acetabular index and medial safe space. Treating physician should be wary of low or negative acetabular index when reaming in THA or when placing screws in anterior internal fixation of the acetabulum.

## Abbreviations

a.p.: Anterior-posterior
AI: Acetabular index
CT: Computed-tomography
MSZ: Medial safe zone
THA: Total hip arthroplasty
